# Manual and educational therapy in the treatment of hemophilic arthropathy of the elbow: a randomized pilot study

**DOI:** 10.1186/s13023-018-0884-5

**Published:** 2018-09-03

**Authors:** Rubén Cuesta-Barriuso, Antonia Gómez-Conesa, José-Antonio López-Pina

**Affiliations:** 1 Department of Physiotherapy, School of Biomedical and Health Sciences, European University of Madrid, Madrid, Spain; 2 Real Fundación Victoria Eugenia, Instituto de Salud Carlos III, 4 Sinesio Delgado Street, 28029 Madrid, Spain; 3 Fishemo CEE-Federación Española de Hemofilia, Madrid, Spain; 4 Research Group in Physiotherapy and Health Promotion, Regional Campus of International Excellence “Campus Mare Nostrum”, University of Murcia, Murcia, Spain; 5 Department of Basic Psychology and Methodology, Faculty of Psychology, University of Murcia, Murcia, Spain

**Keywords:** Elbow, Joint disease, Hemophilia, Physiotherapy modalities

## Abstract

**Background:**

Elbow arthropathy is characteristic in patients with hemophilia. Arthropathy is manifested by decreased range of motion, pain, loss of strength and muscular atrophy, and axial changes. The objective is to evaluate the safety of two physiotherapy programs combining manual therapy and home exercises with educational sessions in patients with hemophilic elbow arthropathy.

**Methods:**

This is a randomized study with 27 patients with elbow hemophilic arthropathy with a mean age of 34.48 (SD: 12.99) years, were randomised to Manual Therapy group, educational group and control group. The physiotherapy programmes were: manual therapy through joint traction, passive muscles stretching and proprioceptive neuromuscular facilitation; and educational sessions and daily home exercises. The study lasted for twelve weeks, with two sessions a week in manual therapy group and one session every two weeks with daily home exercises in educational group. The variables measured were range of motion of elbow, biceps strength, circumference of arm, and elbow pain.

**Results:**

The treatment with manual therapy improved the circumference of arm, flexion elbow and elbow pain. Six months later, MT group still enjoyed improved. In the educational group there were improvements, but not significant, in the measured variables.

**Conclusion:**

Neither of the two physiotherapy interventions caused elbow hemarthrosis. The treatment with manual therapy improved the range of movement and circumference of arm, and lessened pain in hemophilic patients with chronic elbow arthropathy. No hemarthrosis was recorded during treatment or during the follow-up period. Larger randomized clinical trials are required to confirm the results of this study.

**Trial registration:**

(NCT02198040). Registered 22 July 2014, retrospectively registered.

**Electronic supplementary material:**

The online version of this article (10.1186/s13023-018-0884-5) contains supplementary material, which is available to authorized users.

## Background

Hemophilia is a congenital coagulopathy characterized by a deficit or absence of clotting factors. The lacking factor may be factor VIII (FVIII) or factor IX (FIX) and depending on this there are two types of hemophilia (hemophilia A and hemophilia B, respectively) [1]. The circulating percentage of this factor in deficit defines the degree of severity of the hemophilia: severe (< 1%), moderate (1–5%) or mild (> 20%) [2].

Although this disease is hematological, the main clinical manifestations are musculoskeletal (hematomas and hemarthrosis) [3]. Joints bleeds are the main hemorrhagic complication in these patients. Repeated hemorrhagic episodes in a single joint trigger a joint degeneration process (hemophilic arthropathy) [4]. This lesion typically presents decreased range of motion and muscle strength, proprioceptive alterations, chronic pain, and biomechanical and axial changes. Prophylactic pharmacological treatment with intravenous FVIII / FIX concentrates has shown to be the only effective therapeutic option to control hemorrhages and long-term sequelae in the joints [5].

Around 80–90% of patients with hemophilia without primary prophylaxis treatment present elbow arthropathy [6]. This is due, among other causes, to the role played by the upper limbs as ancillary appendages for ambulation, when patients suffer bleeding in the lower limbs. Aid from the upper limbs is manifested in the transition from sitting to standing and by their supporting functions (acting as support or partial support of body weight by means of walking sticks, crutches or walkers) [7].

A typical characteristic of hemophilic arthropathy of the elbow is hypertrophy of the radial head. The state of hyperemia around the open epiphysis in the immature skeleton favors this disproportionate growth. Thus, alteration of the radius limits the movements of forearm pronation and supination [8]. The elbow is not a weight-bearing joint, and early limitations of range of motion (flexion and extension) rarely interfere with functionality [9]. As the joint deterioration progresses the humerus-ulnar joint becomes affected, limiting flexion and extension movements. In this way, the normal development of the routine activities of daily life is affected. In some cases, bone deformity may lead to ulnar nerve neuropathy [7].

Sandfordet et al. [10] observed that up to 67% of patients with hemophilic elbow arthropathy requiring the continued use of a splint showed a low or no adherence to use thereof, making it difficult to achieve the expected improvement with orthotic treatment. The surgical treatment of elbow arthropathy includes synovectomies, simple radial head excision combined with joint debridement, excisional arthroplasty, arthrodesis and interpositional arthroplasty [11]. Radial head excision combined with synovectomy has shown to be effective in improving pain, bleeding frequency and mobility (pronation and supination) [12].

Despite the prevalence of hemophilic arthropathy of the elbow, there are currently no studies based on manual physiotherapy for the treatment of this arthropathy. Although Heijnen and de Kleijn [13] reported improvements in pain and joint mobility following a manual physiotherapy intervention, further studies using a more ambitious methodology are needed.

This study aims to evaluate the safety of two physical therapy programs combining manual therapy and home exercises with educational sessions in patients with hemophilic arthropathy of the elbow.

## Methods

### Study design

A single blind randomized study was conducted with two treatment groups and one control group. Patients included in the experimental groups received physical therapy treatment, namely manual therapy (MT group) and educational therapy (EG group).

### Ethical approval and consent

This study was approved by the Ethics Committee of the University of Murcia, Spain (registration number 43/2011). Prior to the start of the study, all patients signed an informed consent document according to the Declaration of Helsinki 1975, subsequently revised in 2008. Moreover, the study was registered in the International Registry (NCT02198040).

### Data collection and participants

The inclusion criteria for participation in the study were: patients over 18 years-old; medically diagnosed with hemophilia A or B; and diagnosed with hemophilic arthropathy of the elbow in one or both elbows (scoring at least 3 points on the Pettersson X-Ray scale) [14]. Patients excluded from the study were those: diagnosed with other congenital coagulopathies (e.g., von Willebrand disease); who had developed antibodies to FVIII or FIX (inhibitors); and who failed to sign the informed consent document. Patients who for any reason had elbow hemarthrosis during the study period would be excluded.

Throughout the study, patients continued with the same pharmacological treatment regimen with FVIII / FIX concentrates, as previously prescribed by their hematologist (prophylactic or on demand).

**Fig. 1:**
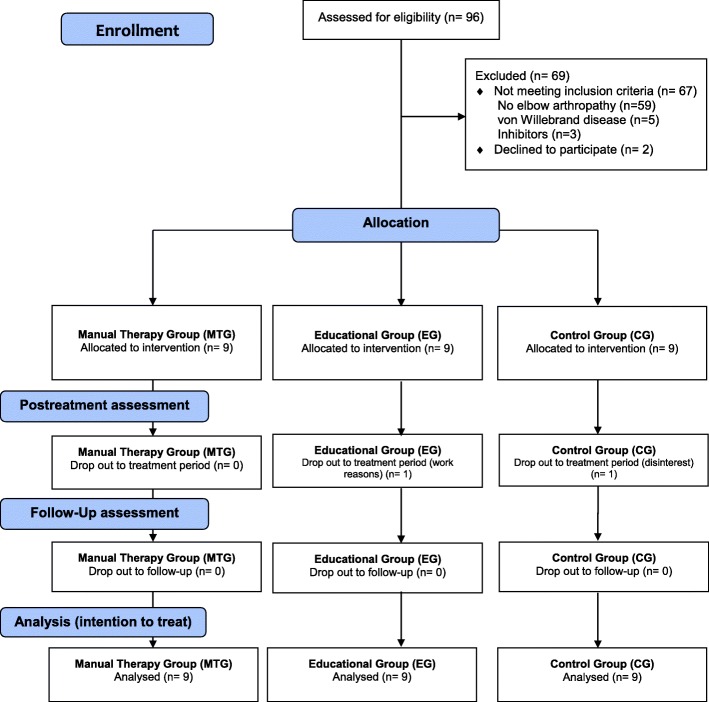
CONSORT Flow Diagram of the study (Additional file 1)

Hemophilia patients treated at the Arrixaca University Hospital of Murcia (Spain) participated in this study. Of the 96 patients with congenital coagulopathies treated at this hospital, 37 had a medical diagnosis of elbow arthropathy. Three patients were excluded due to the development of factor VIII inhibitors and 5 were diagnosed with von Willebrand disease. Of the 29 patients who met the inclusion criteria, 2 patients declined to participate in the study because they lived more than 100 km away from the location where the experimental work was to be performed. Figure 1 shows the study flowchart (Fig. 1).

An administrative assistant, unrelated to the objectives of the study, carried out patient allocation to each of the groups. Randomization, prior to the beginning of the treatment period, was performed using an opaque envelope system, whereby each patient was included in a group sequentially. Thus, 9 patients were included in each of the 3 study groups.

### Intervention

The intervention lasted for 12 weeks, with a regularity of two sessions weekly for the experimental group receiving manual therapy (MT), and one session every two weeks and daily home sessions for the educational physiotherapy group (EG). The content of both experimental treatments is detailed in Table 1.

**Table 1:** Treatment characteristics of the experimental groups

Group	Duration	Intervention	
MT	5 min	Thermotherapy shallow to 50 cm away from the elbow, using a bulb of 250w.	
	15 min	Joint traction of elbow, in submaximal mobility amplitude with distal fixation of humerus and proximal fixation of radius and ulna in neutral position of forearm. Joint traction in I-II degree of flexion and extension submaximal of elbow.	
	15 min	Muscle stretching (within the limits of mobility). Compression technique, passive muscle stretching and relaxation in biceps and triceps.	
	15 min	Proprioceptive neuromuscular facilitation (PNF) of upper limb, from the abduction, flexion and external rotation of shoulder with extension of elbow and dorsal flexion of wrist, to adduction, internal rotation of shoulder with flexion of elbow and palmar flexion of wrist and fingers.	
	10 min	Local cryotherapy with ice bag and protection between it and the skin	
	Duration	Session	Intervention
EG	30 min	1	Theory: Introduction to haemophilia: clinic and treatment. Anatomy and biomechanics of elbow
	20 min		Theory: exercises for the maintenance and improvement of ROM, in favor of gravity
	20 min		Practice: exercises in favor of gravity
	20 min		Resolution of questions and group discussion
	30 min	2	Theory: Anatomy of elbow musculature. Function of muscles and hematomas’ treatment
	20 min		Theory: exercise for maintaining and improving strength
	20 min		Practice: isometric and isotonic exercises of elbow
	20 min		Resolution of questions and group discussion
	30 min	3	Theory: hemarthrosis, synovitis and arthropathy: clinical manifestations and treatment
	20 min		Theory: treatment of pain and mobility
	20 min		Practice: active exercises for mobility and pain management
	20 min		Resolution of questions and group discussion
	30 min	4	Theory: Proprioception: definition and importance in haemophilia
	20 min		Theory: proprioception exercises
	20 min		Practice: elbow proprioception exercises
	20 min		Resolution of questions and group discussion
	30 min	5	Theory: Physical activity and sport: risks and benefits
	20 min		Theory: recommended sports in haemophilia
	20 min		Practice: swimming technique
	20 min		Resolution of questions and group discussion
	30 min	6	Theory review
	30 min		Review of practical exercises
	20 min		Resolution of questions and group discussion

Each session for the patients included in the MT group lasted 60 min. This physiotherapy program included thermotherapy and cryotherapy techniques (to relax the muscles and avoid local swelling at the beginning and end of each session, respectively). Similarly, joint-traction techniques were applied following the manual therapy criteria described by Kaltenborn [15], as well as specific muscle stretching (using the compression-stretching-relaxation technique in manual orthopedic therapy) and proprioceptive neuromuscular facilitation techniques (in order to stretch and strengthen the muscles of the upper limbs).

The treatment performed in the other experimental group consisted of six 90-min sessions every two weeks (including theoretical and practical modules from an educational perspective), together with daily home exercises lasting 20–30 min. The theoretical sessions included pedagogical material regarding relevant clinical aspects of hemophilia (anatomy, clinical aspects of acute and chronic lesions in hemophilia and treatment thereof, and physical activity and sport).

At the end of the theoretical part, patients were taught physiotherapy exercises aimed at improving previously treated aspects, to be carried out at home. In the register of activities that had to be filled in by the patients included in the educational group, we observed a moderate adherence to treatment (62%). Based on a daily register completed by the patients, performance of the home exercises was assessed. These exercises, performed twice a day, were structured as follows: 2 sets of 10 repetitions, each lasting 20 s, with a 10-s rest between sets. This record was collected every two weeks (when the patient received the next session of educational physiotherapy). The program included muscle stretching and isometric exercises of biceps and triceps, and proprioceptive exercises on quadruped’s position with visual support (later, they were performed without visual support).

Finally, no physiotherapy intervention was performed in the control group (CG) and patients continued with their usual routine.

### Variables and measuring instruments

A physiotherapist with experience in the treatment of patients with hemophilia, blinded to patient allocation and study objectives, carried out the assessments. This evaluator conducted the various measurements of the dependent variables under the same conditions. On the other hand, an experienced physiotherapist with more than 30 years of hemophilia-related practice blindly evaluated the radiologic deterioration of the elbows evaluated in the study using the Pettersson X-Ray scale.

The dependent variables analyzed in the study were range of motion, arm perimeter, muscle strength and pain perception.

- Safety of the intervention. At the beginning of the treatment, a record was given to patients, where they were to specify hemorrhagic episodes (muscular and articular) suffered during treatment and follow-up, indicating the symptoms and signs, date and specific location.
- Range of movement. Elbow flexion and extension were measured using a universal goniometer. The anatomical references used were those provided by Querol [16], using the zero-reference method for the moving arm of the goniometer as noted by Norkin &White [17].
- Arm perimeter. The muscle perimeter was assessed following the protocol described by Querol [16]. A tape measure was placed in the middle of the muscular belly of the biceps brachii.
- Biceps brachii muscle strength. In order to quantify muscle strength in both muscles, the rupture test for patients with hemophilia was used, following the evaluation criteria described by Querol [16]. Test scores range from 0 to 5 points (0 indicates normal muscle strength while 5 is no contraction whatsoever).
- Perception of joint pain. To evaluate the pain caused by hemophilic arthropathy, we used the visual analog scale (VAS). This scale, which has been widely used in hemophilia, scores from 0 to 10 points (0 indicates no pain and 10 the maximum pain imaginable for the patient). Each patient was asked to report the perception of joint pain during the previous month.

To measure the degree of damage to the elbow joint at baseline, the Pettersson X-Ray scale was used [14]. This measuring instrument evaluates radiological joint deterioration and consists of 8 items and scores from 0 to 13 points (wherein 0 indicates that there is no joint damage and 13 the maximum damage of the elbow).

Patients were evaluated three times: before the intervention, at the end of treatment, and after a 6-month follow-up period.

Prior to the start of the study, a pilot trial was performed to determine the inter-rater reliability in the measurement of range of motion, arm perimeter and muscle strength. The physical therapist who acted as the study evaluator and a physiotherapist with experience in hemophilia carried out the evaluations. This pilot test included 10 subjects without any kind of elbow pathology. There was a high inter-rater reliability in the measurement of dependent variables (*p* < 0.01), with significant inter-rater correlations in arm circumference (intraclass = 1.00), flexion (intraclass = 0.96) and elbow extension (intraclass = 0.94), and muscle strength of the biceps brachii (intraclass = 0.64).

### Statistical analysis

Statistical analysis was performed using the statistical package SPSS version 19.0 for Windows (IBM Company, Armonk, NY, USA). The descriptive statistics of the various study variables (before and after treatment, and after the follow-up period) were calculated. Inter-rater reliability was obtained with intraclass correlation.

In order to analyze the equality between groups, an ANOVA and the non-parametric Kruskal-Wallis test were applied. The Student’s t-test for paired samples and the Wilcoxon non-parametric test were used to compare the dependent variables means in the three groups.

In this study, an intention-to-treat analysis was performed. Due to multiple group comparisons in this study design there is an increased in the type 1 error rate, therefore significance level for the post-hoc analysis was 0.017, and the effect size was calculated using the Cohen formula [18].

## Results

The median age of the 27 patients included in the study was 34.48 years (IQR = 12.99), with a median weight of 81.60 kg. (IQR = 9.71). Most patients had a diagnosis of hemophilia A (81.4%), had a severe phenotype (62.9%) and were receiving prophylactic replacement therapy with clotting concentrates at the time of the study (55.5%). Regarding the clinical characteristics of the evaluated elbows with hemophilic arthropathy, the median number of hemarthrosis during the year prior to the start of the study was 1.00 hemarthrosis (IQR = 1.25), presenting a median elbow radiological deterioration of 9.00 points (IQR = 4.25). When analyzing the equality between the three groups at the beginning of the study, we observed that there were no differences in most of the dependent variables. Significant differences were only observed in the elbow pain variable (*p* = 0.003). Table 2 shows the main clinical and anthropometric characteristics of the patients included according to the allocation to the different study groups.

**Table 2:** Descriptive characteristics of patients (*n* = 27) and their elbows with hemophilic arthropathy (*n* = 46), at baseline in each group of the study

Variables	Group MT			Group EG			Group C			Sig.
	*n*	*Medians*	*IQR*	*n*	*Medians*	*IQR*	*n*	*Medians*	*IQR*	
Age of patient (years)	9	28.00	13.25	9	32.00	34.00	9	37.50	27.00	.719
Weight of patient (Kg)	9	85.20	12.57	9	71.40	15.67	9	82.35	7.80	.179
Elbows hemarthrosis in the previous year (number)	16	1.00	0.75	16	1.00	1.00	14	0.00	2.00	.541
Radiological joint deterioration (0–13)	16	10.0	2.75	16	8.50	6.75	14	7.00	3.25	.133
Strength (0–5)	16	0.00	0.00	16	0.00	0.00	14	0.00	0.00	.306
Circumference (cm)	16	30.75	4.23	16	31.00	4.47	14	31.25	5.40	.774
Flexion (degree)	16	141.0	17.00	16	147.0	29.50	14	149.00	20.00	.926
Extension (degree)	16	6.00	18.50	16	12.0	36.50	14	0.00	15.00	.208
Pain (0–10)	16	0.50	1.00	16	0.00	0.37	14	0.00	0.125	.003
	n	%		n	%		n	%		
Type (Hemophilia A / Hemophilia B)	6 / 3	66.7 / 33.3		8 / 1	89.1 / 10.9		8 / 1	89.1 / 10.9		.404
Severity (Severe / Mild)	8 / 1	89.1 / 10.9		6 / 3	66.7 / 33.3		3 / 6	33.3 / 66.7		.494
Treatment (Prophylaxis / On demand)	7 / 2	77.8 / 22.2		6 / 3	66.7 / 33.3		2 / 7	22.2 / 77.8		.841

*MT* manual therapy group, *EG* educational group, *CG* control group, *n* number of patients, *IQR* Interquartile range; %: percentage

When analyzing the changes after the treatment period in each group, we observed that there were only changes in the patients treated with manual therapy. In these patients there was improvement in and pain perception (*p* = 0.006), and also a marginally significant improvement in elbow flexion (*p* = 0.022) and the perimeter of the arm (*p* = 0.050).

No differences were observed during this period in the patients who carried out physical therapy exercises at home and the control group patients. After the follow-up period, improvements observed in the manual therapy group were maintained (*p* = 1.00 and *p* = 0.069, in flexion and elbow pain, respectively), without detecting any other changes in the remaining groups. Table 3 shows the changes in the different assessments.

**Table 3:** Medians (and Interquartile range) of physical measurements and pain perception, of the elbows with hemophilic arthropathy evaluated in the different assessments and analysis after the treatment and follow up period

Group	Variables	Post-treatment				Follow up			
		Medians (IQR)	Sig.	W	ES	Medians (IQR)	Sig.	W	ES
MT	Strength (0–5)	0.00 (0.00)	.188	.180	−.34	0.00 (0.00)	.188	1.00	.00
	Circumference (cm)	30.85 (3.75)	.050	.018	.11	30.70 (4.37)	.113	.098	−.23
	Flexion (degree)	143.00 (13.25)	.022	.040	.28	146.00 (10.25)	1.00	.972	.00
	Extension (degree)	5.00 (19.50)	.293	.278	.07	4.00 (22.00)	.553	.562	−.03
	Pain (0–10)	0.00 (0.875)	.**006**	.**014**	**−.49**	0.00 (0.00)	.069	.068	−.50
EG	Strength (0–5)	0.00 (0.00)	.333	.317	.00	0.00 (0.00)	.333	.317	.25
	Circumference (cm)	31.05 (5.175)	.484	.145	.03	30.45 (5.62)	.268	.279	−.06
	Flexion (degree)	144.50 (36.75)	.121	.121	−.17	148.00 (28.25)	.101	.114	.21
	Extension (degree)	12.00 (39.25)	.528	.734	−.02	10.50 (29.75)	.090	.090	−.16
	Pain (0–10)	0.00 (0.00)	.164	.157	−.20	0.00 (0.375)	.270	.257	.46
CG	Strength (0–5)	0.00 (0.00)	.165	.157	−.19	0.00 (0.00)	^a^	1.00	^a^
	Circumference (cm)	31.35 (5.90)	.687	.476	−.03	30.75 (7.55)	.687	.859	−.04
	Flexion (degree)	146.50 (11.25)	.664	.944	.02	142.00 (11.75)	.712	.610	−.01
	Extension (degree)	0.00 (11.75)	.666	1.00	−.02	0.00 (5.00)	.534	.715	−.03
	Pain (0–10)	0.00 (0.00)	.165	.157	−.23	0.00 (0.00)	.752	.785	.13

*MT* manual therapy group, *EG* educational group, *CG* control group, *n* number of elbows, *SD* standard deviation, *Sig*. signification, *W* non-parametric Wilcoxon test, *ES* effect size), ^a^No can be calculated the signification and the correlation because the standard error of the difference is 0

## Discussion

Extension is the first movement of the elbow joint that becomes limited in cases of hemophilic arthropathy of the elbow [19]. After treatment, however, we observed a marginally significant improvement in elbow flexion in the patients included in the group treated with manual therapy. Few studies have analyzed the efficacy of manual therapy in hemophiliac arthropathy, focusing especially on ankle arthropathy [20, 21]. Although Heijnen and de Kleijn [13] were the first to observe how, after a 5-year period, elbow mobility was maintained in patients with hemophilia treated using articular traction, to date there were no studies evaluating a specific treatment protocol. In our study, we noted that during the follow-up period, improvement of elbow flexion was maintained, without any bleeding episodes whatsoever. The functional range of the elbow is between 75° and 120° [22]. Although we found no differences in elbow extension, improved flexion may be a first step in improving elbow functionality in these patients.

Although there were no changes in biceps muscle strength in any of the study groups, we observed a marginally significant improvement related to the arm circumference of patients treated with manual therapy. These findings in connection with the arm perimeter are in agreement with those achieved by Gomis et al. [23] after an 8-week intervention using electrostimulation. In the Valencian sample it was noted how the diameter of the isometric strength and the electromyographic activity of the biceps brachii increased in patients with hemophilic arthropathy of the elbow. The elbow mobility of the patients included in the educational group did not improve. One of the possible causes could be the moderate adherence to the realization of domiciliary exercises. In patients with hemophilia, adherence to prophylactic treatment has been observed [24] greater than in other chronic diseases. However, the absence of well-defined protocols, clear scientific evidence and their implementation in the different treatment centers, indicates in the usual clinical practice a low-moderate adherence in the performance of domiciliary exercises, as we have observed in the patients of the educational group included in our study.

Pain has shown to be a major cause of disability in patients with hemophilia, affecting functional capacity and their perception of quality of life [25]. After treatment, an improvement in elbow pain was observed in patients allocated to the manual therapy group. As in our study results, applying joint tractions and manual therapy has already proved effective in the treatment of hemophilic arthritis pain [13, 20, 21].

During our study, no patients needed to be excluded due to the development of elbow hemarthrosis. This absence of elbow hemarthrosis during treatment and follow-up in both treatment groups compares favorably against the incidence of hemarthrosis in patients during the previous year. Therefore, it is fair to say that both treatments, applied appropriately, are safe. This same incidence of bleeding has been observed in studies based on manual therapy [13, 20, 21] and educational interventions and home exercises [26].

Although educational physiotherapy interventions have shown to be effective in parents of children with hemophilia [27] and in combination with home exercises in adults with hemophilic arthropathy [26], improvements in the elbow joint were not observed. The sample size or the duration of the intervention might explain the insignificant results. Another factor to consider is that the elbow joint is not a load-bearing joint, and in variables such as pain in subjects who do not require technical aids, it is more difficult to observe differences.

### Limitations to the study

The main limitation is the low sample size. Although hemophilia is a disease with a low prevalence and 73% of patients with elbow arthropathy have been recruited from the Virgen de la Arrixaca Hospital, the sample is limited. However, in the absence of physiotherapy studies focused on this joint, this study may serve as an approximation to the goal of obtaining evidence in the treatment of this effect from hemophilia.

Another limitation of the present study is the use of common measuring instruments. Although the study tools (VAS, goniometry, Daniels scale, etc.) are used in the clinical and hospital approach to patients, the use of more specific and objective techniques would have given greater validity and consistency to the results obtained.

### Future lines of research

Although the elbow is not a load-bearing joint and from the biomechanical point of view, it is less invalidating than the ankle or the knee, it is essential to investigate the treatment of elbow arthropathy.

Carrying out randomized clinical trials confirming the safety and efficacy of manual therapy treatment as discussed in this study could prove the suitability of this technique in patients with hemophilia.

Similarly, through studies with a large sample size and over a considerable period of time, the role of suitable educational physiotherapy interventions in adult hemophilia patients should be researched.

The extension is the movement of elbow previously affected by the development of hemophilic arthropathy, while pronation and supination are functionally very affected in these patients. However, we have not observed changes in these movements after the intervention in the manual therapy group. Future studies should focus on the development of manual therapy protocols aimed at facilitating the mobility of the radio head and pronation-supination. Although the development of a hypertrophy of the head of the radius, osteophytes or a significant reduction of the joint space, considerably limits the possibilities of improving the range of motion, it can act on structures whose mobility is still reversible (musculature, joint capsule and connective tissue).

## Conclusions

Two Physical Therapy programs, based on manual therapy and home exercises to improve range of motion, muscle strength and proprioception, may be considered safe, as they did not cause elbow hemarthrosis during the study period.

A physiotherapy treatment through joint traction, muscle stretching, and proprioceptive neuromuscular facilitation may improve flexion and pain perception in patients with hemophilic arthropathy of the elbow. These results can be maintained over a period of 6 months.

Randomized clinical trials with a larger sample of patients with hemophilic arthropathy of the elbow are recommended, using the physiotherapy programs described here in to confirm the results obtained in this study.

## Additional file

Additional file 1: Supplementary data. (DOCX 14 kb)

## Abbreviations

ANOVA: Analysis of variance
FIX: factor IX
FVIII: factor VIII
SD: standard deviation
VAS: visual analog scale
