# Supplementary material for: Manual and educational therapy in the treatment of hemophilic arthropathy of the elbow: a randomized pilot study
Source: Orphanet J Rare Dis. 2018 Sep 3;13:151. doi: 10.1186/s13023-018-0884-5 (PMC6122620; doi:10.1186/s13023-018-0884-5)
Supplement: Supplementary file 1 — Supplementary data. (DOCX 14 kb) [file 13023_2018_884_MOESM1_ESM.docx]

What this paper adds?

- Hemophilia is a rare hematological disease that is characterized by musculoskeletal bleeding and incapacitating sequels that affects not only physically and the perception of quality of life and social relationships of these patients
- In a relatively small sample of patients with hemophilia, this study suggests the safety of Physiotherapy techniques to prevent hemarthrosis and the functional improvement of the elbow.
- Physiotherapy treatments can help to improve the perception of pain in patients with hemophilic arthropathy of the elbow.
- The functional improvement of the elbow can help to improve disabling squeals of hemophilic arthropathy and with it social relationships of these patients.
- This is the first clinical study that includes a physiotherapy intervention through orthopedic manual therapy in the treatment of patients with hemophilic elbow arthropathy.
